# Childhood physical abuse in outpatients with psychosomatic symptoms

**DOI:** 10.1186/1751-0759-2-8

**Published:** 2008-03-21

**Authors:** Masanori Handa, Hideyuki Nukina, Masako Hosoi, Chiharu Kubo

**Affiliations:** 1 Tachiarai Hospital, 842-1 Yamaguma, Chikuzen-machi, Asakura-gun, Fukuoka 838-0823, Japan; 2 Mitsubishi Kobe Hospital, 6-1-34 Hyogo-ku, Kobe 652-0863, Japan; 3 Department of Psychosomatic Medicine, Graduate of Medical Sciences, Kyushu University, 3-1-1 Maidashi, Higashi-ku, Fukuoka 812-8582, Japan

## Abstract

**Background:**

In Japan and Asia, few studies have been done of physical and sexual abuse. This study was aimed to determine whether a history of childhood physical abuse is associated with anxiety, depression and self-injurious behavior in outpatients with psychosomatic symptoms.

**Methods:**

We divided 564 consecutive new outpatients at the Department of Psychosomatic Medicine of Kyushu University Hospital into two groups: a physically abused group and a non-abused group. Psychological test scores and the prevalence of self-injurious behavior were compared between the two groups.

**Results:**

A history of childhood physical abuse was reported by patients with depressive disorders(12.7%), anxiety disorders(16.7%), eating disorders (16.3%), pain disorders (10.8%), irritable bowel syndrome (12.5%), and functional dyspepsia(7.5%). In both the patients with depressive disorders and those with anxiety disorders, STAI-I (state anxiety) and STAI-II (trait anxiety) were higher in the abused group than in the non-abused group (p < 0.05).

In the patients with depressive disorders, the abused group was younger than the non-abused group (p < 0.05). The prevalence of self-injurious behavior of the patients with depressive disorders, anxiety disorders and pain disorders was higher in the abused groups than in the non-abused groups (p < 0.005).

**Conclusion:**

A history of childhood physical abuse is associated with psychological distress such as anxiety, depression and self-injurious behavior in outpatients with psychosomatic symptoms. It is important for physicians to consider the history of abuse in the primary care of these patients.

## Background

Reports of abuse and domestic violence have been increasing in Japan. However, few studies have been done of the prevalence of physical or sexual abuse history in Japan and Asia. In a large, national telephone survey of 1,145 men and 1,481 women in the United States, Finkelhor et al. reported the prevalence of child sexual abuse history to be 27% for women and 16% for men [1]. The American rate of physical abuse history has been estimated at 5.7 cases per 1000 children [2]. Drossman and Leserman reported a high prevalence of sexual and physical abuse history among female outpatients referred to a gastroenterology clinic [3]. They found that 44% of the studied women reported some type of sexual and/or physical abuse history. Since this report, there has been increasing interest in the relationship between a history of abuse and gastrointesitinal (GI) symptoms. Women with sexual abuse history were reported to have more pain, non-GI somatic symptoms, psychological distress and functional disability compared to those without sexual abuse history, and women with physical abuse history also had a worse health outcome on most health status indicators [4]. Many persistently ill patients, not only those with GI disorders but also patients with psychiatric disorders seeking psychosomatic primary care, have been abused. In Japan and Asia, few studies have examined the health impact of physical and sexual abuse. We have been studying the psychological pain mechanism of patients with functional gastrointestinal disorders (FGID) [5,6]. In our research and treatment of FGID patients, we have found a number of victims of childhood physical abuse.

We focused on a history of childhood physical abuse in outpatients not only with FGID but also with other somatic or psychiatric disorders. We hypothesized a history of abuse would be associated with anxiety, depression and self-injurious behavior, and therefore studied the history of childhood physical abuse in outpatients with psychosomatic symptoms to determine the relationship.

## Methods

### Participants

Enrolled participants were 564 consecutive outpatients examined on their first visit to the Department of Psychosomatic Medicine of Kyushu University Hospital from May 2001 to May 2002. All patients completed a self-reported questionnaire about a history of childhood physical abuse and self-injurious behavior. The self-reported questionnaires were given to all new patients on their first visit to our clinic and needed to be answered quickly and without hesitation before a clinical interview. We divided the patients into two groups: a childhood physically abused group and a non-abused group. Psychological test scores and the prevalence of self-injurious behavior history were compared between the two groups. All participants gave informed written consent before entering the study.

### Definition

A structured clinical interview of the patients and a psychiatric diagnosis was done according to DSM IV [7]. For the FGID diagnosis, irritable bowel syndrome (IBS) and functional dyspepsia (FD) were defined by the Rome II criteria [8].

### Questionnaire

A history of childhood physical abuse was surveyed using a modified, self-reported questionnaire by Leserman and Drossman [9,10]. A history of self-injurious behavior was queried in a similar manner (Table 1).

**Table 1:** Questionnaire concerning childhood physical abuse and self-injurious behavior

1. When you were child, did an older person hit, kick, or beat you?	(yes, no)
2. Have you ever injured and/or intended to injure yourself?	(yes, no)

### Psychological Assessment

All patients completed a validated, self-reported questionnaire for anxiety [State Trait Anxiety Inventory (STAI-I is the state anxiety scale and STAI-II is the trait anxiety scale [11])] and one for depression [Zung self-rating depression scale (ZSDS)[12]].

### Statistical Methods

Mann-Whitney U test, Fisher's exact test, and unpaired t-test (two tailed) were used to compare age, gender, the duration of disease, and the psychological test score (STAI-I, STAI-II, ZSDS) with the prevalence of self-injurious behavior as a psychiatric symptom.

## Results

Of the 564 outpatients, 518 had psychiatric disorders such as depressive disorders (n = 323), anxiety disorders (n = 60), eating disorders (n = 98) [anorexia nervosa (n = 37), bulimia nervosa (n = 42), others (n = 19)], pain disorder (n = 37). 46 outpatients had mainly somatic symptoms not categorized in psychiatric disorders. 88 were FGID patients including 48 IBS patients and 40 FD patients, 34(70.8%) IBS patients and 22(55%) FD patients had psychiatric disorders such as a depressive disorder or an anxiety disorder. 14 patients with somatic disorders other than FGID and without psychiatric disorders had no experience of childhood physical abuse. A history of childhood physical abuse was reported by 71 of 518 patients with such psychiatric disorders (13.7%); 41 of 323 patients with a depressive disorder (12.7%), 10 of 60 patients with an anxiety disorder (16.7%), 16 of 98 patients with an eating disorder (16.3%), 4 of 37 patients with a pain disorder (10.8%)(Table 2). In FGID patients a history of childhood physical abuse was reported by 6 of 48 IBS patients (12.5%), and 3 of 40 FD patients (7.5%) (Table 3).

**Table 2:** Characteristics and psychological test (STAI-I, II, ZSDS) scores of patients with psychiatric disorders

**Psychiatric disorder (DSM-IV)**	**Abused**	**Non-abused**	**p-Value**
**depressive disorders (n = 323)**	**n = 41**	**n = 282**	
**Age (years) **†	36 (29,42.5)	41.5 (30,56)	0.0428 (a)
**female [N (%)]**	29 (70.7)	176 (62.4)	0.3858 (b)
**Duration of disease (months) **†	12 (3,27.5)	8 (3,24)	0.5501 (a)
STAI-I*	61.5 ± 8.6	57.2 ± 10.2	0.0107 (c)
STAI-II*	62.3 ± 9.3	57.5 ± 11.8	0.0042 (c)
ZSDS*	55.7 ± 7.8	53.3 ± 8.8	0.0991 (c)
**self-injury [N (%)]**	25 (61)	71 (25.2)	p < 0.0001 (b)

**anxiety disorders (n = 60)**	**n = 10**	**n = 50**	
**N(%)**			0.0020 (d)
**panic disorder**	2 (20)	31 (62)	
**posttraumatic stress disorder (PTSD)**	6 (60)	6 (12)	
**social phobia**	2 (20)	13 (26)	
**Age (years) **†	26 (18.5,29.5)	32 (24,37)	0.1078 (a)
**female [N (%)]**	9 (90)	29 (58)	0.0762 (b)
**Duration of disease (months) **†	15 (1,102)	7 (1,36)	0.4793 (a)
STAI-I*	65.7 ± 6.4	51.6 ± 11.0	p < 0.0001 (c)
STAI-II*	64.8 ± 9.4	52.1 ± 13.3	0.0057 (c)
ZSDS*	54.2 ± 12.7	47.3 ± 10.5	0.0720 (c)
**self-injury [N (%)]**	8 (80)	7 (14)	0.0001 (b)

**eating disorders (n = 98)**	**n = 16**	**n = 82**	
**N (%)**			0.2299 (d)
**anorexia nervosa**	3 (18.8)	34 (41.5)	
**bulimia nervosa**	9 (56.3)	33 (40.2)	
**other eating disorders**	4 (25)	15 (18.3)	
**Age (years) **†	23 (18.25,29.75)	22 (17,27.5)	0.6032 (a)
**female [N (%)]**	16 (100)	79 (96.3)	1.0000 (b)
**Duration of disease (months) **†	30 (10.5,71.5)	25 (7,72)	0.6969 (a)
STAI-I*	56.3 ± 11.3	54.9 ± 9.7	0.6085 (c)
STAI-II*	58.8 ± 13.9	58.6 ± 11.4	0.9508 (c)
ZSDS*	54.6 ± 10.1	54.3 ± 8.9	0.9042 (c)
**self-injury [N (%)]**	11 (68.8)	42 (51.2)	0.2746 (b)

**pain disorder (n = 37)**	**n = 4**	**n = 33**	
**Age (years) **†	39.5 (35.25,49.75)	47 (30,57.5)	0.5405 (a)
**female [N (%)]**	1 (25)	22 (66.7)	0.1419 (b)
**Duration of disease (months) **†	28.5 (7.5,54.75)	14 (7.5,57.5)	0.8256 (a)
STAI-I*	56.0 ± 9.9	52.4 ± 9.1	0.4634 (c)
STAI-II*	57.0 ± 13.3	51.2 ± 12.9	0.4028 (c)
ZSDS*	48.5 ± 10.3	46.6 ± 10.3	0.7297 (c)
**self-injury [N (%)]**	4 (100)	4 (12.2)	0.0011 (b)

* The results are expressed as mean ± SD.

†The results are expressed as medians (25%,75%).

p-Values estimated by Mann-Whitney U test (a), Fisher's exact test (b), unpaired t-test (two tailed) (c) or chi-square test with Yates's correction (d)

**Table 3:** Characteristics and psychological test (STAI-I, II, ZSDS) scores of patients with functional gastrointestinal disorder

	**Abused**	**Non-abused**	**p-Value**
**irritable bowel syndrome (n = 48)**	**n = 6**	**n = 42**	
**Age (years) **†	28 (25,34.75)	31.5 (19.75,39.5)	0.6966 (a)
**female [N (%)]**	4 (66.7)	17 (40.5)	0.3827 (b)
**Duration of disease (months)**†	10.5 (6.5,75)	16 (6.75,40.5)	0.9130 (a)
STAI-I*	59.5 ± 6.6	56.7 ± 8.7	0.4541 (c)
STAI-II*	67 ± 5.8	56.6 ± 12.0	0.0047 (c)
ZSDS*	53 ± 10.5	53.5 ± 9.1	0.9021 (c)
**self-injury [N (%)]**	2 (33.3)	13 (31)	1.0000 (b)

**functional dyspepsia (n = 40)**	**n = 3**	**n = 37**	
**Age (years) **†	31 (27,39)	37 (24,51.5)	0.7775 (a)
**female [N (%)]**	2 (66.7)	20 (54.1)	1.0000 (b)
**Duration of disease (months)**†	42 (3,96)	20 (8.5,73.5)	0.8572 (a)
STAI-I*	60 ± 8.2	54.2 ± 12.7	0.4445 (c)
STAI-II*	59 ± 5.3	54.7 ± 13.3	0.3167 (c)
ZSDS*	54.7 ± 7.5	50.1 ± 8.9	0.3910 (c)
**self-injury [N (%)]**	0 (0)	4 (10.8)	1.0000 (b)

* The results are expressed as mean ± SD.

†The results are expressed as medians (25%, 75%).

p-Values estimated by Mann-Whitney U test (a), Fisher's exact test (b), unpaired t-test (two tailed) (c) or chi-square test with Yates's correction(d)

In both the patients with depressive disorders and those with anxiety disorders, STAI-I (state anxiety) and STAI-II (trait anxiety) were higher in the abused groups than in the non-abused groups (p < 0.05). In the patients with depressive disorders, the abused group was significantly younger than the non-abused group (p < 0.05) (Table 2). 6 of 10 abused patients with anxiety disorders (60%) had posttraumatic stress disorder (PTSD). 31 of 50 non-abused patients with anxiety disorders (62%) had a panic disorder. No significant differences were found between the abused groups and the non-abused groups in terms of both the duration of the disease and the percentage of females (Table 2, 3). The prevalence of self-injurious behavior history of the patients with depressive disorders, anxiety disorders and pain disorders was higher in the abused groups than in the non-abused groups (p < 0.005) (Table 2). Of the FGID patients, only the IBS patients had higher STAI-II (trait anxiety) scores in the abused group than in the non-abused group (p < 0.005) (Table 3).

## Discussion

Childhood abuse represents a serious international problem, with abuse victims at high risk of depression, suicide and behaviors such as substance abuse and running away from home [13,14]. Childhood sexual or physical abuse is a great risk factor for self-cutting and suicide attempts [15,16]. It was reported in Japan that subjects with habitual self- mutilation more frequently had a history of suicide attempts, sexual abuse, and childhood physical abuse than general psychiatric controls[17]. We found childhood physical abuse influenced younger patients with depression (Table 2).

High rates of both childhood sexual (27%) and physical abuse and of eating disturbance (20% to 25%) have been reported in the general adult female population of the United States [18]. Physical abuse history was associated with high dissociation in female patients with eating disorders in Japan [19]. Our study showed no significant difference in the development of eating disorders between the physically abused group and the non-abused group (Table 2). Although we did not examine sexual abuse in these patients, we have often during treatment found patients with eating disorders who had been sexually abused.

Rapkin et al. showed that physical abuse history was more prominent than sexual abuse history in female patients with chronic pelvic pain [20]. Previous sexual abuse history is a significant predisposing risk for somatization and non-somatic chronic pelvic pain [21]. Patients with chronic pelvic pain showed a high prevalence of depression, substance abuse, adult sexual dysfunction, somatization, and history of childhood and adult sexual abuse [22]. Leserman et al. showed that women with a history of abuse were much more likely to report somatic symptoms associated with panic, depression, musculoskeletal disorders, and genito-urinary disorders [23]. In our study there were no significant differences between the physically abused group and the non-abused group in the percentage of females, however we have also experienced many outpatients with psychiatric disorders who reported somatic complaints at their first visit.

A history of abuse is found to be common in FGID patients seen at referral centers in comparison to primary care clinics [24]. In research on the relation between GI symptoms and a history of abuse in a US study, of 130 subjects with IBS, 56 (43.1%) had experienced sexual abuse and 8 (6.2%) had suffered physical abuse, and of 200 subjects with dyspepsia, 70 (35%) had experienced sexual abuse and 9(4.5%) had suffered physical abuse [25]. Our study showed higher rates of childhood physical abuse history in patients with FGID (12.5% of IBS, 7.5% of FD) than a US population-based study (Table 3). The prevalence of physical or sexual abuse history in patients with FGID was reported differently with wide range in different communities [24]. It may be different among psychosocial situations, cultures and races.

The above indicate that childhood physical abuse history strongly influences emotional instability, pain, and behavior. We found a significantly high rate of self-injurious behaviors in the physically abused patients with depressive, anxiety and pain disorders. In both the patients with depressive and anxiety disorders, STAI-I and II (state and trait anxiety) were higher in the abused groups than in the non-abused groups (p < 0.05) (Table 2). 60% of abused patients with anxiety disorders had PTSD, while 62% of non-abused patients with anxiety disorders had a panic disorder. This difference seems to show that strong psychological effects from abuse may result from stress-effects on controlling areas of the brain [26]. Childhood abuse in the setting of PTSD has been reported to be associated with long-term changes in brain structure (e.g. smaller hippocampal volume) and function by neuroimaging studies [27].

The relationship between childhood abuse, self-injury and borderline personality disorder has been reported [28], however the outpatients in our study had no borderline personality disorder. The resultant psychological trauma associated with childhood physical abuse might induce self-injurious behaviors such as cutting and suicide attempts. Childhood physical abuse seems to be associated with interpersonal violence or personality dysfunction [29]. The number of abused patients with FGID in our study may be too small to evaluate more exactly the difference between the abused group and the non-abused group. However, the high score on STAI-II of the abused group of IBS patients seems to suggest a link between childhood trauma and trait anxiety, which influences bowel dysfunction (Table 3) [30,31]. A history taking of childhood physical abuse indicating childhood trauma induced by poor family or social functioning is useful for the primary treatment with patients with psychosocial symptoms.

This study has some methodological limitations. We studied referrals to a university- based tertiary care clinic of psychosomatic medicine in an urban area of Japan. The prevalence of childhood physical abuse history in our study also might be higher than that of population-based studies in Japan. A simple yes-no, self-report screening questionnaire about only childhood physical abuse history was used. When we conducted a clinical interview of patients after their questionnaires were finished, we did not always focus on a history of abuse in the psychiatric diagnosis. This question was not done by an interview, so there is concern about the reliability for stating an adverse health outcome from only physical abuse. In Drossman's first study, 11 of the 12 physically abused patients were also sexually abused. Almost one third of the abused patients had never discussed their experiences with anyone and only 17% had informed their doctor [3]. Leserman et al. stressed the relationship between abuse severity (e.g. life threatening physical abuse) and somatic symptoms [23]. However, we dared not ask about the frequency or degree of physical abuse which would be life-threatening. It might be difficult to recognize quickly the difference between physical abuse and physical punishment as discipline from an older person. It is also possible that the result of the prevalence of a history of physical abuse in our study might be more excessively estimated than that of the true physical abuse. However, the prevalence of not only physical abuse but sexual abuse in the community is uncertain in different studies in North America [3,32]. Even in the population based US study, a similar self-report screening questionnaire was mailed to a random sample of persons, and physical abuse was reported substantially less often [25]. This study was not only for research but for primary patient care. Sexual abuse, which has been reported to be the strongest measure of abuse, was not determined in this study. Some victims of sexual abuse were probably among our patients. The prevalence of self-injurious behavior of the patients with eating disorders and FGID was not higher in the childhood physical abused groups than the non-abused groups. It is also possible that more victims of sexual abuse might be among the patients with eating disorders and FGID. We respect and keep the right to privacy of our patients. Questions about sexual abuse should only be asked in a structured interview using valid and reliable measures [4,10] and after the patient-therapist relationship has been well established.

## Conclusion

Our study showed a history of childhood physical abuse was associated with anxiety, depression and self-injurious behavior in outpatients with psychosomatic symptoms. Determining the possibility of a history of abuse is necessary to formulate a treatment plan for new outpatients with psychosomatic complaints. Future population-based research including both physical and sexual abuse will be necessary to further this line of study. Further cross-cultural psychosocial studies on abuse need to be globally promoted.

## Competing interests

The author(s) declare that they have no competing interests.

## Authors' contributions

M. Handa designed the study, analyzed and interpreted the data, and drafted the manuscript. HN participated in its design and coordination and helped draft the manuscript. M. Hosoi and CK supported the design and coordination of the study. All authors read and approved the final manuscript.
